# Supplementary material for: A glycosylated lipooctapeptide promotes uptake and growth of Mycobacterium abscessus in the host
Source: Nat Commun. 2025 Apr 8;16:3326. doi: 10.1038/s41467-025-58455-5 (PMC11978893; doi:10.1038/s41467-025-58455-5)
Supplement: Supplementary file 1 — Supplementary Information [file 41467_2025_58455_MOESM1_ESM.pdf]

Supplementary Information

A glycosylated lipooctapeptide promotes uptake and growth of *Mycobacterium abscessus* in the host

Louis David Leclercq, Vincent Le Moigne, Wassim Daher, Mélanie Cortes, Bertus Viljoen, Yara Tasrini, Xavier Trivelli, Hélène Lavanant, Isabelle Schmitz-Afonso, Nicolas Durand, Franck Biet, Yann Guérardel, Laurent Kremer, Jean-Louis Herrmann

Table of content

Supplementary figures

Fig. S1 p.2
Fig. S2 p.4
Fig. S3 p.6
Fig. S4 p.8
Fig. S5 p.10
Fig. S6 p.12
Fig. S7 p.14

Supplementary tables

Table S1 p.16
Table S2 p.17

Raw NMR and MS data are freely available at https://zenodo.org/records/14918825.

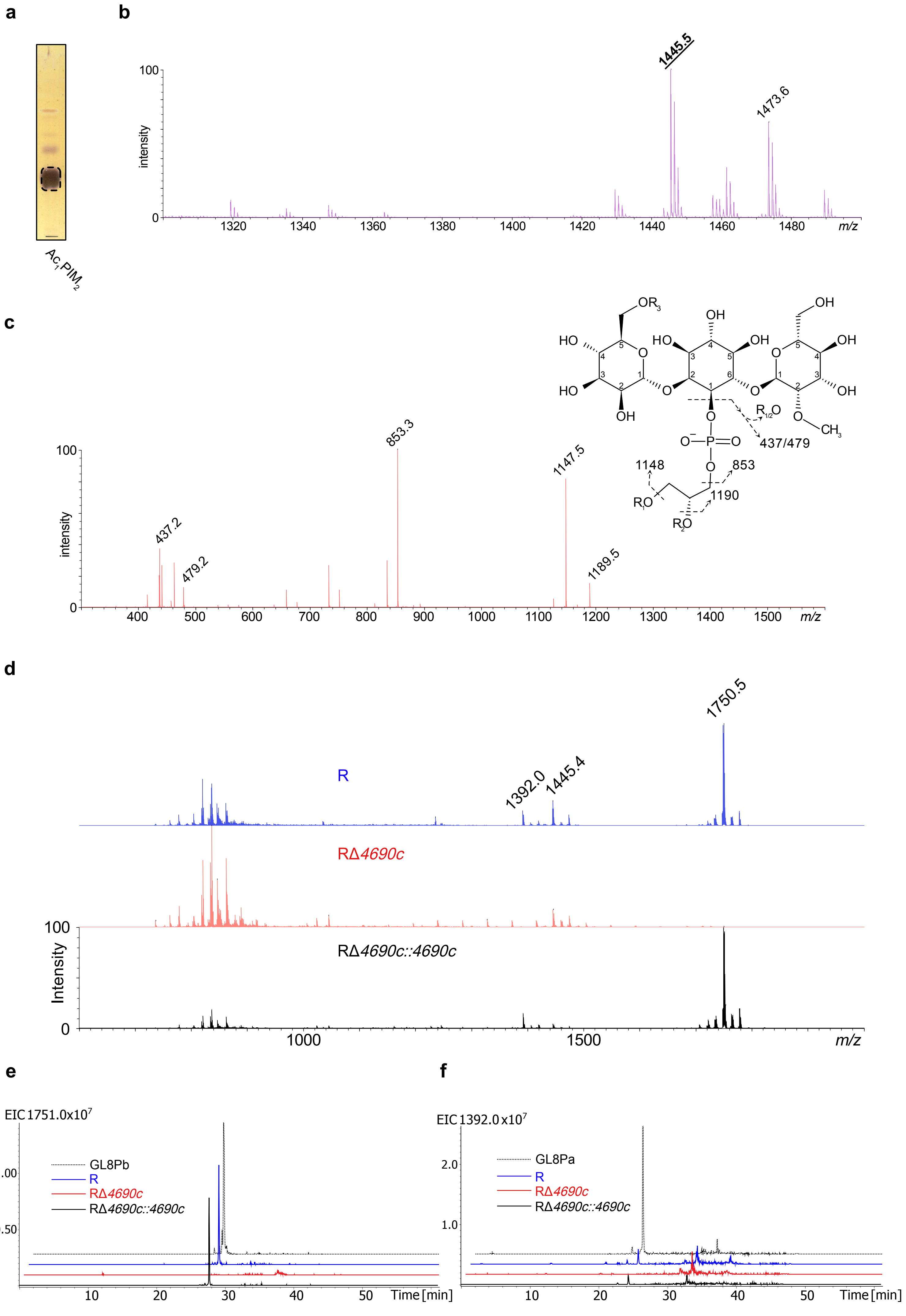
a
b
c
d
e
f
Ac1PIM2
intensity
100
0
1445.5
1473.6
1320
1340
1360
1380
1400
1420
1440
1460
1480
m/z
853.3
1147.5
437.2
479.2
1189.5
400
500
600
700
800
900
1000
1100
1200
1300
1400
1500
OR3
OH
HO
O
CH3
R1/2O
O−
P=O
437/479
1148
853
1190
R1O
R2O
1750.5
1392.0
1445.4
R
RΔ4690c
RΔ4690c::4690c
Intensity
1000
1500
EIC 1751.0x10^7
1.00
0.50
GL8Pb
10
20
30
40
50
Time [min]
EIC 1392.0 x10^7
2.0
1.0
GL8Pa

Fig. S1. Lipid analysis of *M. abscessus* R, R△*4690c*, and R△*MAB_4690c::4690c*. TLC (**a**) MALDI-MS (**b**) and MALDI-MS^2 (**c**) analyses of Ac_1PIM_2 purified from *M. abscessus* R polar lipids confirmed the attribution of $[M-H+2Na]^+$ adduct at *m/z* 1446 with R_1 as C_{19} tuberculostearic, R_2 as C_{16} palmitic acid and R_3 as myristic acid while a 2-*O*-methyl mannose and a mannose are linked to the inositol. **(d)** MALDI-MS spectra in positive mode of polar lipids from R (blue) and R△*MAB_4690c::4690c* (black) show two signals at *m/z* 1392 (GL8Pa) and 1750 (GL8Pb), which are absent in R△*4690c* (red). Conversely, ion at *m/z* 1446 corresponding to $[M-H+2Na]^+$ adduct of Ac_1PIM_2 was detected in all strains grown on LB. Extracted ion chromatograms at *m/z* 1392 (**e**) and 1750 (**f**) of polar lipids isolated from *M. abscessus* R (blue), R△*4690c* (red), and R△*MAB_4690c*::*4690c* (black) grown on LB show that GL8Pa and GL8Pb (dashed lines) are not detected in R△*4690c* by LC-MS.

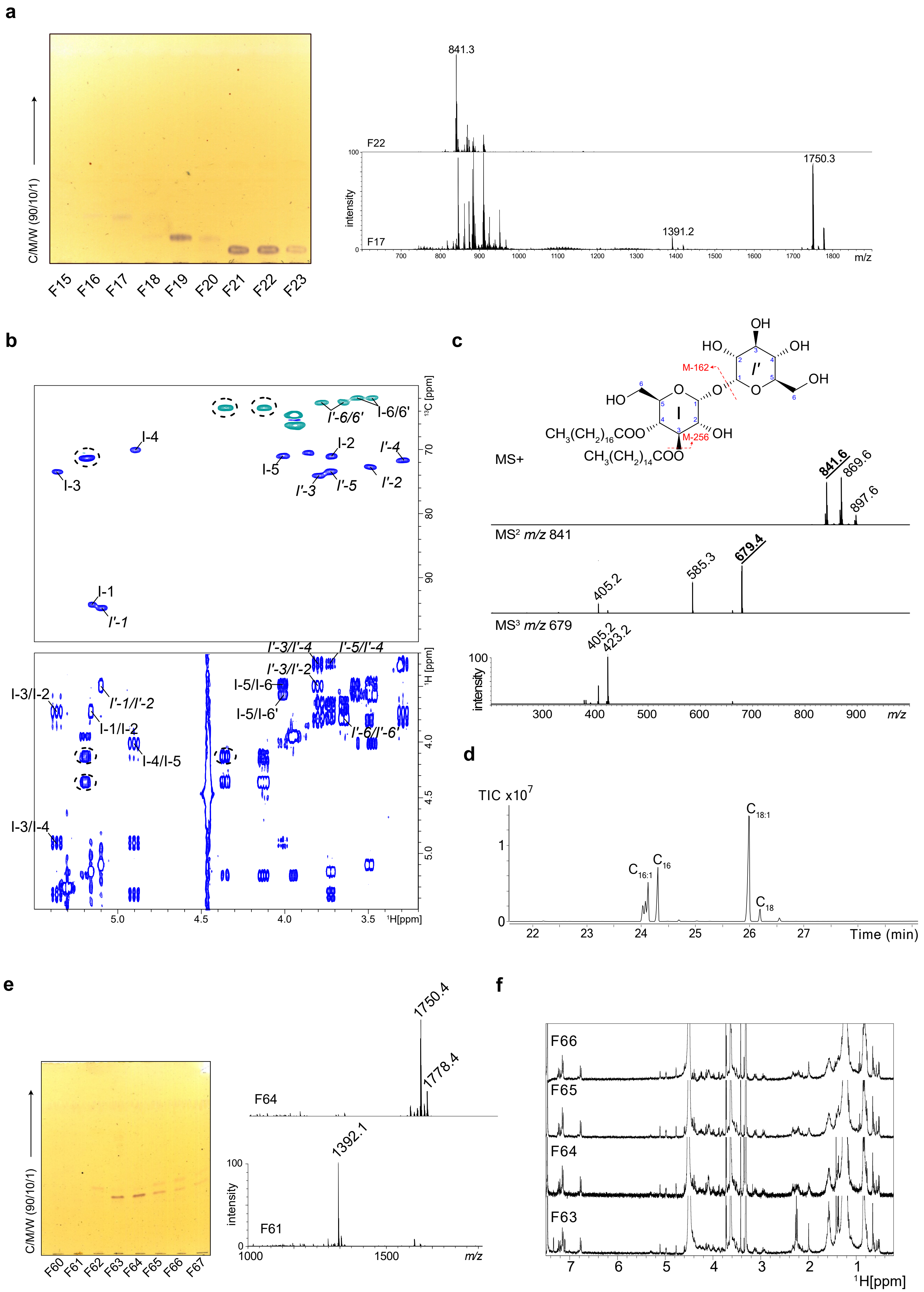
a
C/M/W (90/10/1)
F15 F16 F17 F18 F19 F20 F21 F22 F23
841.3
F22
F17
1391.2
1750.3
intensity
m/z
b
I-3
I-4
I-5
I-2
I'-3
I'-5
I'-2
I'-4
I'-6/6'
I-6/6'
I-1
I'-1
13C [ppm]
1H [ppm]
1H[ppm]
I-3/I-2
I'-1/I'-2
I-1/I-2
I-4/I-5
I-3/I-4
I'-3/I'-4
I'-5/I'-4
I'-3/I'-2
I-5/I-6
I-5/I-6'
I'-6/I'-6'
c
MS+
841.6
869.6
897.6
MS2 m/z 841
405.2
585.3
679.4
MS3 m/z 679
405.2
423.2
intensity
m/z
M-162
M-256
CH3(CH2)16COO
CH3(CH2)14COO
HO
OH
I
I'
d
TIC x10^7
C16:1
C16
C18:1
C18
Time (min)
e
C/M/W (90/10/1)
F60 F61 F62 F63 F64 F65 F66 F67
1750.4
1778.4
F64
1392.1
F61
intensity
m/z
f
F66
F65
F64
F63
1H[ppm]

Fig. S2. Purification of GL8Pa and GL8Pb. (a) Polar lipids were extracted and separated by increasing concentrations of methanol on silica gel liquid chromatography prior to analysis by TLC and MALDI-TOF. GL8Pa and GL8Pb were observed, by MALDI-TOF MS analysis, as signals at *m/z* 1391.2 and 1750.3, respectively, eluted by 10% methanol (fraction 17). The intense band in fraction 22 that eluted at 15% methanol, was identified as 3,4 di-*O*-acyl trehalose by a combination of high resolution $^{1}H/^{13}C$ HSQC and $^{1}H/^{1}H$ TOCSY NMR (**b**), MS^n fragmentation (**c**), and GC-MS analyses (**d**). Fraction 17 was identified by NMR as a glycerophospholipid (data not shown). **(e)** An additional purification step using reverse-phase liquid chromatography removed the glycerophospholipid contaminant and separated GL8Pa (fraction 61) and GL8Pb (fractions 63-66) after elution with 100% methanol, as confirmed by ^{1}H NMR analysis (**f**). Following purification, the structure of GL8Pb (at *m/z* 1750) was resolved by NMR, GC-MS, and mass spectrometry. The signal at *m/z* 1750 was accompanied by a minor signal at *m/z* 1778, tentatively identified as an isoform of GL8Pb with a longer fatty acyl chain (**Fig. S6c**). GL8Pa could not be purified in sufficient quantity to allow complete NMR analysis and was therefore exclusively analyzed by mass spectrometry and compared with the major compound GL8Pb.

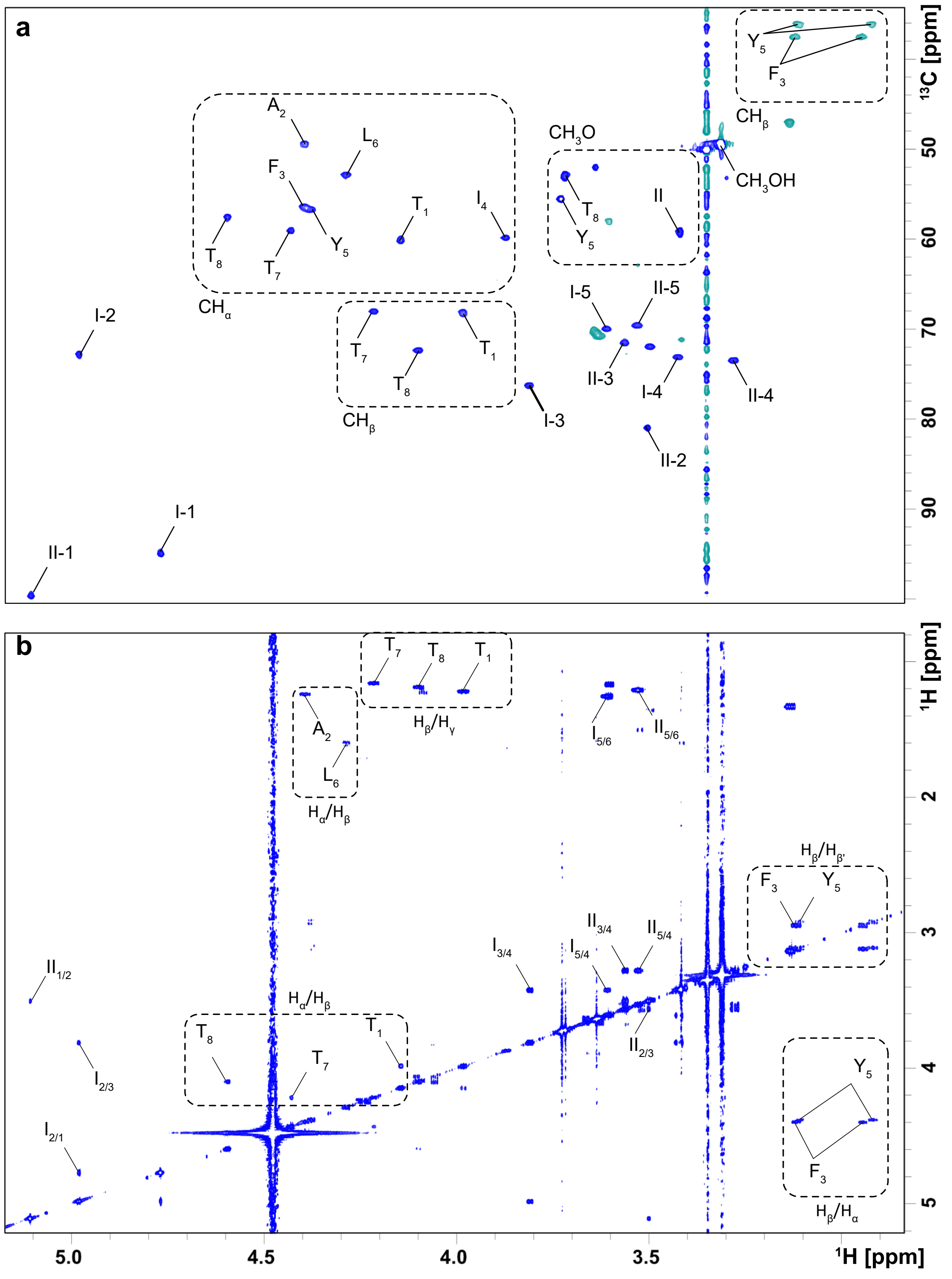
a
Y_5
F_3
CH_β
A_2
L_6
F_3
T_1
I_4
T_8
Y_5
T_7
CH_α
CH_3O
T_8
Y_5
II
CH_3OH
I-2
I-5
II-5
T_7
T_1
T_8
CH_β
I-3
II-3
I-4
II-4
II-2
I-1
II-1
^{13}C [ppm]
50
60
70
80
90
b
T_7
T_8
T_1
A_2
L_6
H_β/H_γ
H_α/H_β
$I_{5/6}$
$II_{5/6}$
^1H [ppm]
2
3
4
5
$H_\beta/H_{\beta'}$
F_3
Y_5
$II_{3/4}$
$II_{5/4}$
$I_{3/4}$
$I_{5/4}$
$II_{1/2}$
H_α/H_β
T_8
T_1
T_7
$II_{2/3}$
$I_{2/3}$
$I_{2/1}$
Y_5
F_3
H_β/H_α
5.0
4.5
4.0
3.5
^1H [ppm]

Fig. S3. NMR analysis of amino-acid and glycan moieties of GL8Pb. $^{1}H/^{13}C$ HSQC (**a**) and $^{1}H/^{1}H$ COSY (**b**) NMR spectra of purified GL8Pb acquired in $CDCl_3/CD_3OD$ (2:1) at 293K. Amino-acids are numbered with single-letter codes from the *N*-terminus to the *C*-terminus. The α and β positions are grouped in dotted boxes, along with methoxy (or carbomethoxy) signals, on the HSQC. On the COSY NMR spectrum, dotted areas highlight correlations between α/β positions, β/β' of phenylalanine and tyrosine, and β/γ positions of threonines. The spin system of Rha-I and Rha-II is also displayed.

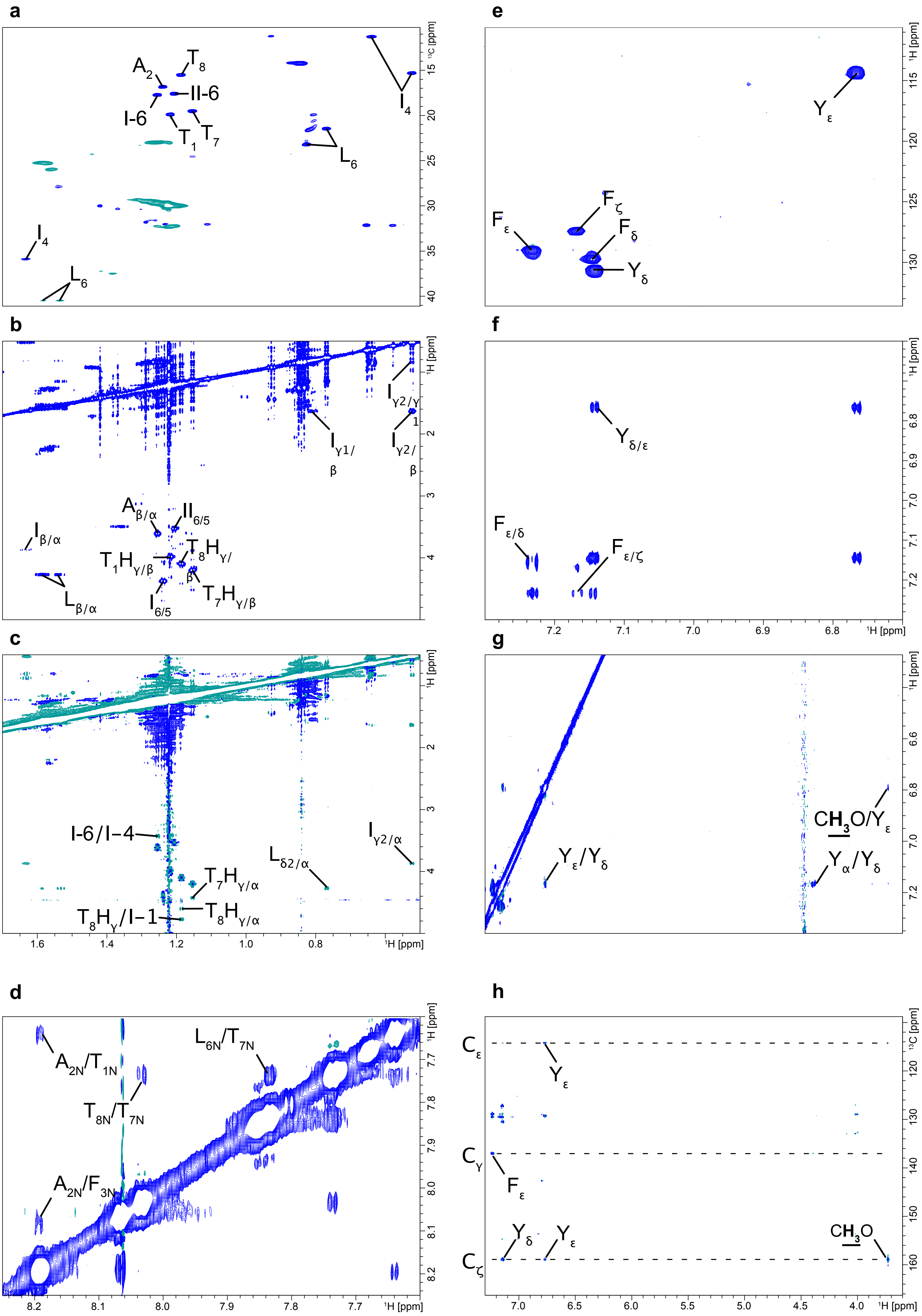
a
b
c
d
e
f
g
h
A2
T8
II-6
I-6
T1
T7
I4
L6
13C [ppm]
1H [ppm]
Aβ/α
II6/5
Iβ/α
T1Hγ/β
T8Hγ/β
Lβ/α
I6/5
T7Hγ/β
Iγ1/β
Iγ2/β
I-6/I-4
T7Hγ/α
T8Hγ/α
T8Hγ/I-1
Lδ2/α
Iγ2/α
A2N/T1N
T8N/T7N
L6N/T7N
A2N/F3N
Yε
Fζ
Fε
Fδ
Yδ
Yδ/ε
Fε/δ
Fε/ζ
CH3O/Yε
Yε/Yδ
Yα/Yδ
Cε
Cγ
Cζ
CH3O

Fig. S4. NMR analysis of amino-acid side-chain of GL8Pb. (a) The $^{1}H/^{13}C$ HSQC NMR spectrum of purified GL8Pb acquired in $CDCl_3/CD_3OD$ (2:1) at 293K displays methyl signals from threonines, aliphatic amino-acids, and rhamnoses. **(b)** Their β/α and γ/β scalar correlations are identified on the $^{1}H/^{1}H$ COSY spectrum. **(c)** The γ/α and δ/α dipolar correlations for threonine, isoleucine, and leucine residues are shown on the $^{1}H/^{1}H$ NOESY NMR spectrum. Two NOESY cross-peaks are detected between C4 and C6 positions of Rha-I, and between the C1 position of Rha-I and the γ proton of Thr-8. **(d)** The $^{1}H/^{1}H$ NOESY NMR spectrum acquired in $CDCl_3/CD_3OH$ (2:1) at 293K shows cross-peaks between the amide protons of Ala-2 and Thr-7 with their neighboring amino-acids. **(e-g)** Analysis of aromatic amino-acids: Phenylalanine aromatic signals $CH_{\delta,\varepsilon,\zeta}$ were identified on the $^{1}H/^{13}C$ HSQC NMR spectrum at 7.15/129.54 ppm, 7.23/128.89 ppm, and 7.17/127.31 ppm, while their $^{1}H/^{1}H$ COSY cross-peaks suggest the presence of a benzyl group (**e-f**). The quaternary carbon at the γ position was identified at 136.9 ppm on the $^{1}H/^{13}C$ HMBC NMR spectrum (**h**). CH_δ and CH_ε of tyrosine were identified on the $^{1}H/^{13}C$ HSQC NMR spectrum at 7.14/130.48 ppm and 6.77/114.19 ppm respectively (**e-f**), whereas the quaternary C_ζ was observed at 158.89 ppm, owing to correlations with CH_δ and CH_ε on the $^{1}H/^{13}C$ HMBC NMR spectrum (**h**). The tyrosine C_ζ also showed a strong correlation on the $^{1}H/^{13}C$ HMBC NMR spectrum with a methoxy group at 3.728/55.31 ppm. The vicinity between CH_δ and CH_ε as well as the methoxy substitution of C_ζ was confirmed by NOESY cross-peaks (**g**).

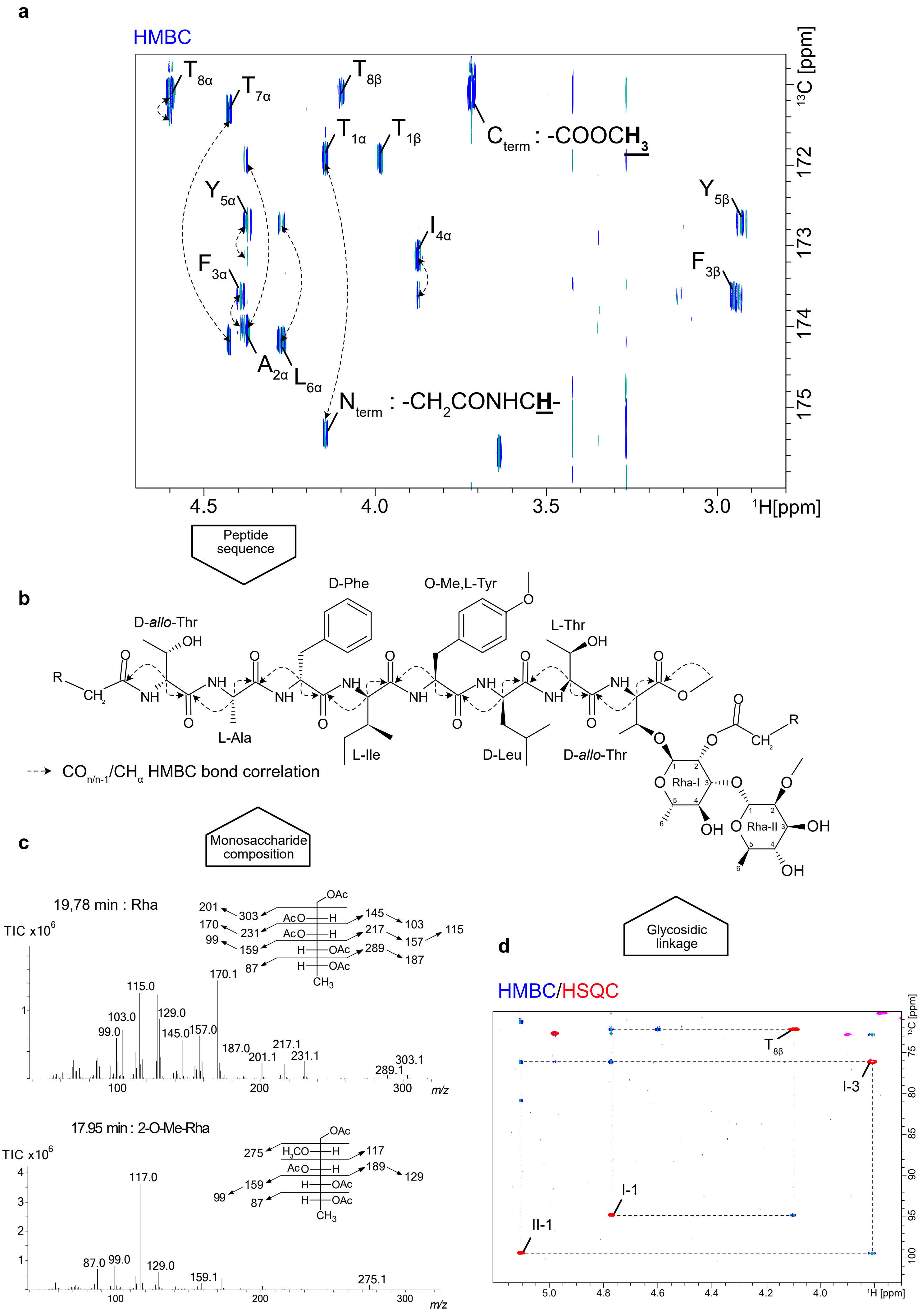

a
HMBC
T8α
T7α
T8β
T1α
T1β
Cterm : -COOCH3
Y5α
Y5β
I4α
F3α
F3β
A2α
L6α
Nterm : -CH2CONHCH-
13C [ppm]
1H[ppm]
Peptide sequence
b
D-allo-Thr
D-Phe
O-Me,L-Tyr
L-Thr
L-Ala
L-Ile
D-Leu
D-allo-Thr
Rha-I
Rha-II
CO n/n-1/CHα HMBC bond correlation
c
Monosaccharide composition
19,78 min : Rha
TIC x10^6
17.95 min : 2-O-Me-Rha
m/z
d
Glycosidic linkage
HMBC/HSQC
T8β
I-3
I-1
II-1

Fig. S5. Total sequencing of GL8Pb. (a) The amino acids sequence of GL8Pb was established by $^{1}H/^{13}C$ HMBC NMR starting from the carboxymethyl group of Thr-8 at 3,717/52,718 ppm at the C-terminal. All α protons and carbonyl groups, as well as some β protons, were sequentially positioned until the sequence was completed with the characterization of the *N*-terminal amino-acid Thr-1. Finally, the α proton of the *N*-terminal amino-acid Thr-1 was shown to correlate with a carbonyl group at 175.29 ppm, strongly suggesting that the N-terminal side of the octapeptide is acylated. **(b)** Correlations of α protons from each amino-acid are highlighted through black dashed arrows along the polypeptidic chain of GL8Pb stereochemical structure. **(c)** Monosaccharide compositional analysis of itol-acetates from purified GL8Pb shows two chromatographic peaks at Tr = 17.95 min and 19.78 min (**Fig. 4d**) identified as 2-*O*-methyl rhamnose and rhamnose respectively, based on electronic impact mass spectrometry analysis (https://glygen.ccrc.uga.edu/ccrc/specdb/ms/pmaa/pframe.html). **(d)** Superimposed $^{1}H/^{13}C$ HSQC and $^{1}H/^{13}C$ HMBC NMR spectra show reciprocal cross-peaks from proton to carbon between Rha I-1 and β Thr-8, as well as between Rha II-1 and Rha I-3 (black dashed lines), confirming the nature of the two bonds.

a

Formula	Exact mass	Sodium adduct	Unsaturations	Δ (mDa)	Δ (ppm)
C75H146N20O25	1727.0768	1750.0660	13	-0.229	-0.13
**C90H150N8O24**	**1727.0763**	**1750.0655**	**20**	**0.274**	**0.16**
C89H25N11O30	1727.0769	1750.0661	83	-0.307	-0.18
C91H146N12O20	1727.0776	1750.0669	25	-1.064	-0.61
C74H150N16O29	1727.0755	1750.0647	8	1.108	0.63
C89H154N4O28	1727.0750	1750.0642	15	1.611	0.92
C87H142N18O18	1727.0749	1750.0642	26	1.621	0.93
C90H21N15O26	1727.0782	1750.0674	88	-1.645	-0.94

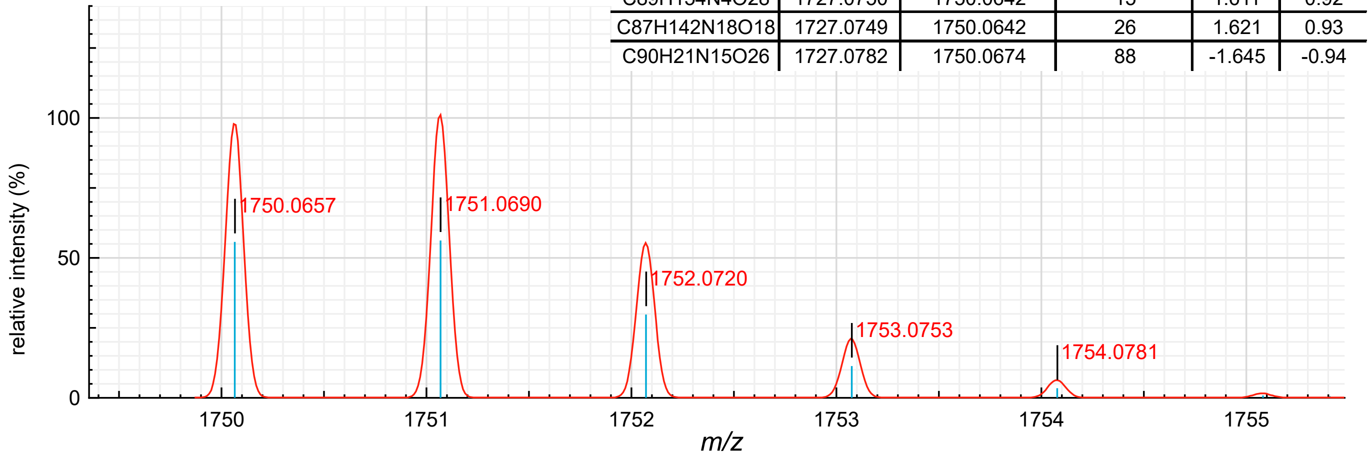

b

Formula	Exact mass	Sodium adduct	Unsaturations	Δ (mDa)	Δ (ppm)
**C71H116N8O18**	**1368.8408**	**1391.8300**	**18**	**0.025**	**0.02**
C100H108N2O2	1368.8411	1391.8303	48	-0.296	-0.21
C58H124N6O29	1368.8413	1391.8305	0	-0.488	-0.35
C87H116O13	1368.8416	1391.8308	30	-0.809	-0.58
C70H120N4O22	1368.8394	1391.8286	13	1.363	0.98

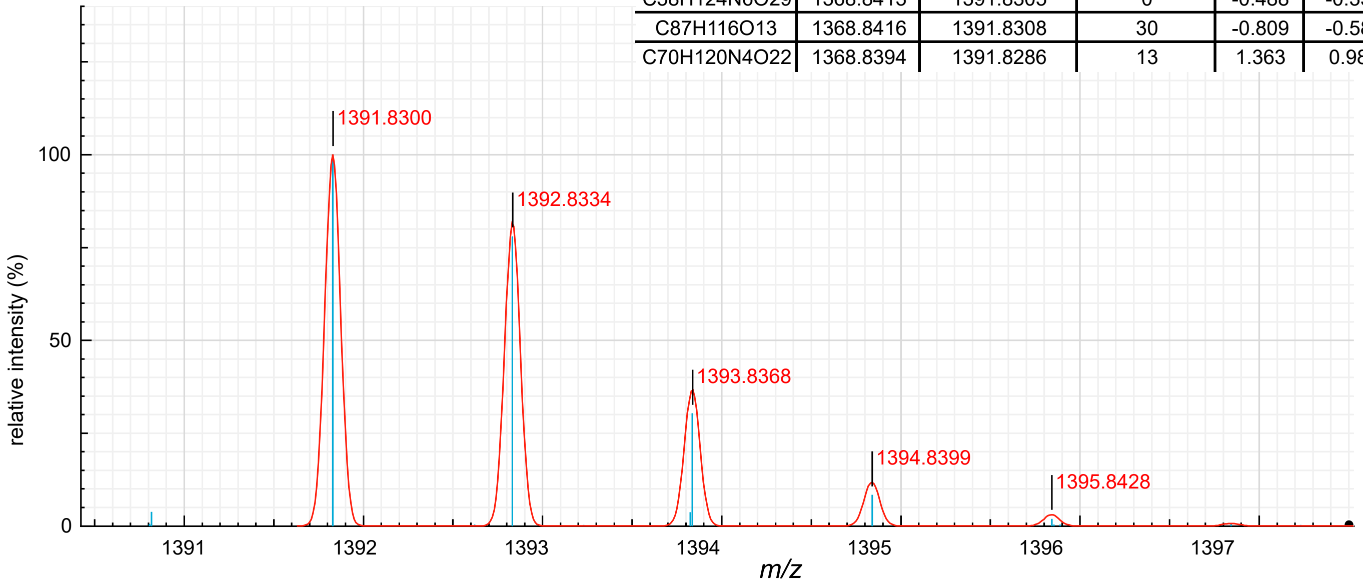

c

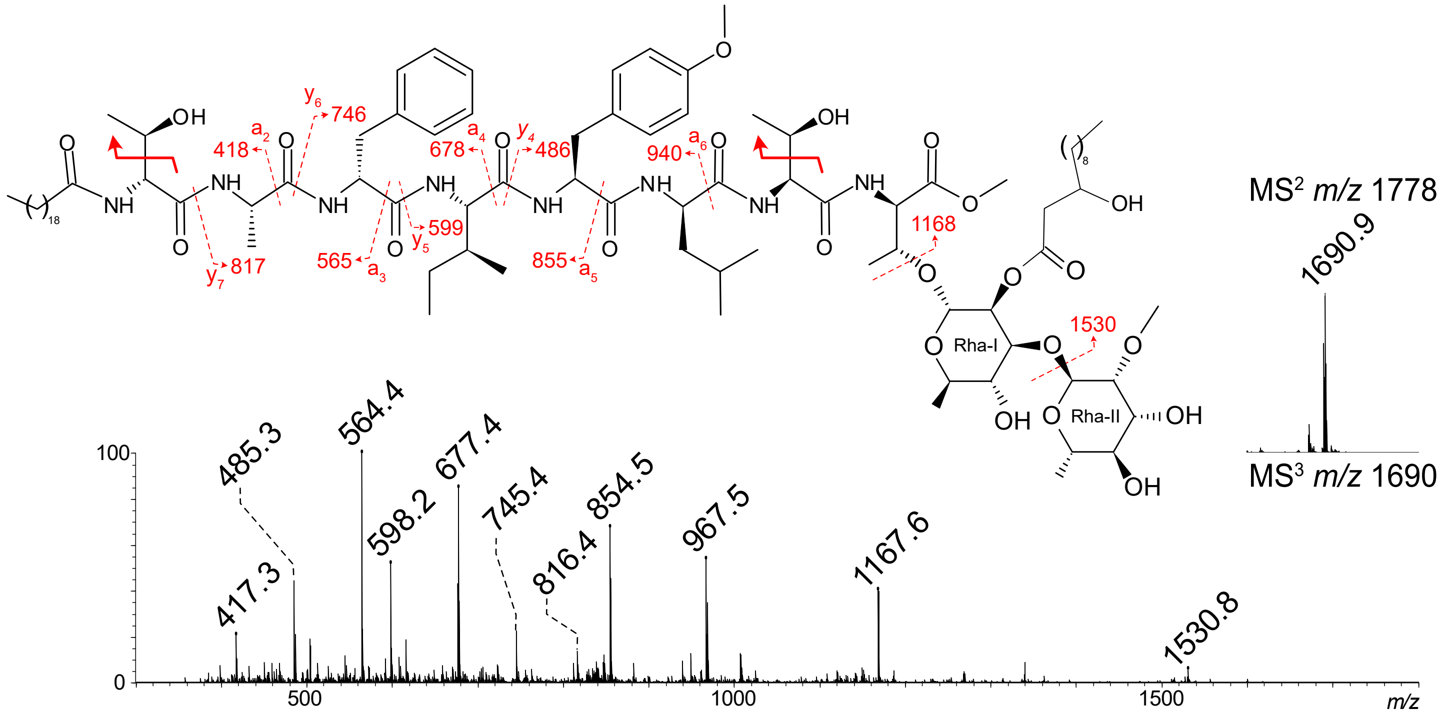

Fig. S6. High resolution mass spectrometry analysis of GL8P variants. Theoretical (red) and measured (blue) isotopic profiles of positive mode MALDI FT-ICR MS^n spectra of GL8Pb (**a**) and GL8Pa (**b**). The tables summarize the atomic composition proposed by the online tool https://ms.epfl.ch/applications/theoretical-calculations/ *via* a query from $C_{0\text{-}100}H_{0\text{-}200}N_{0\text{-}20}O_{0\text{-}30}$ using the measured exact mass of the sodium adduct and a maximal error of 1 ppm. The selected formulas appear in bold. **(c)** The MS^2 fragmentation of the parent ion at *m/z* 1778 generated $[M\text{-}88+Na]^+$ fragment at *m/z* 1690 due to the loss of the two free threonine side chains (red arrows). The MS^3 fragmentation pattern of the ion at *m/z* 1690 was dominated by a_2 to a_6 +28 a.m.u fragments compared to parent ion at 1662 (**Fig. 5a**) while y_4 to y_7 remained the same (red dashed arrows), which suggests that it is substituted by an *N*-arachidonyl group. The loss of the terminal 2-*O*-methyl rhamnose was observed owing to the $[M\text{-}160+Na]^+$ fragment at *m/z* 1530, while the ion at *m/z* 1168 suggests the presence of a 2-*O*-Me-α-rhamnopyranosyl(1-3)2-*O*-hydroxylauryl-α-rhamnopyranose motif.

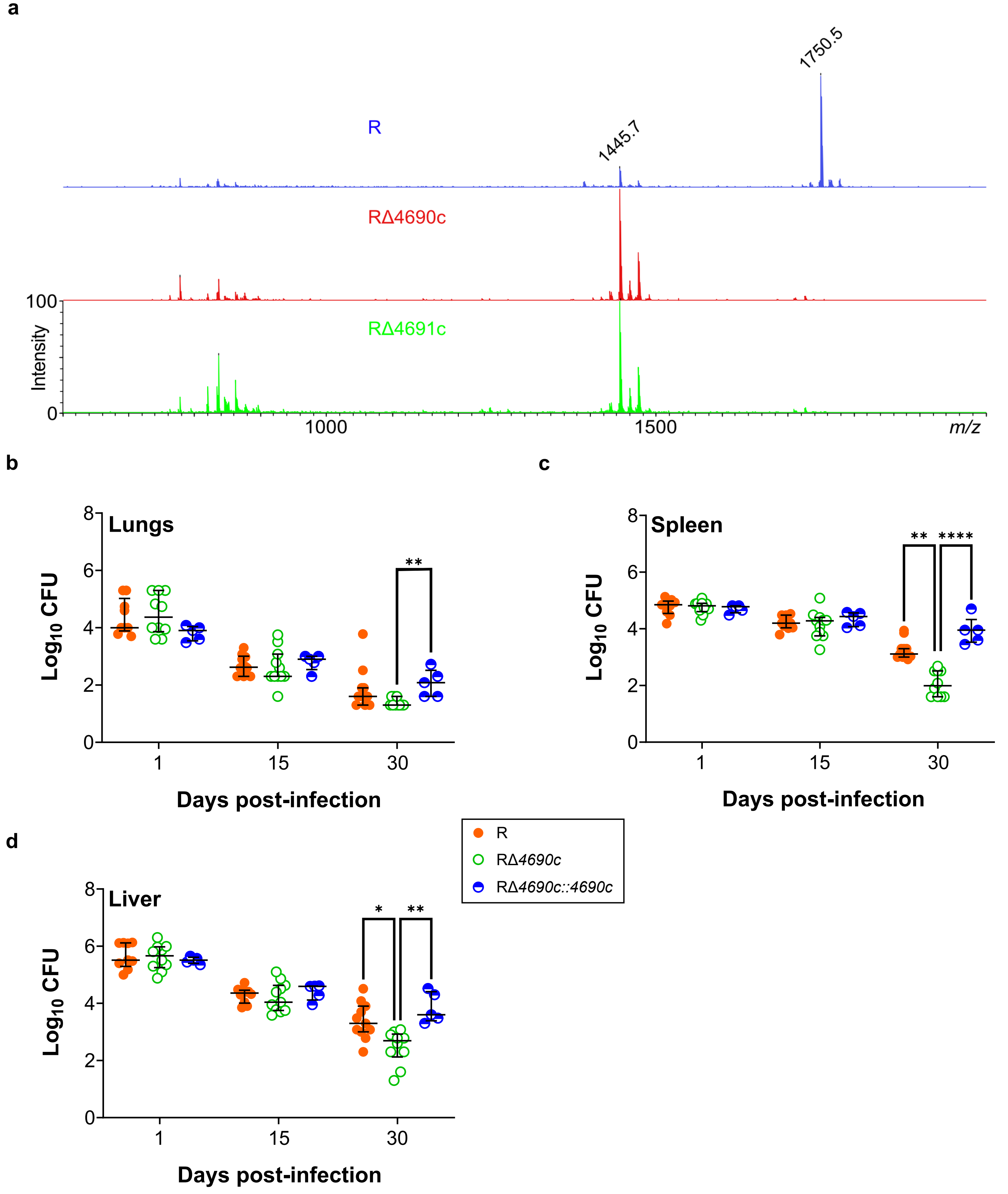
a
R
1750.5
1445.7
RΔ4690c
RΔ4691c
100
Intensity
0
1000
1500
m/z
b
8
Lungs
6
**
Log10 CFU
4
2
0
1
15
30
Days post-infection
c
8
Spleen
**
****
6
Log10 CFU
4
2
0
1
15
30
Days post-infection
d
8
Liver
*
**
6
Log10 CFU
4
2
0
1
15
30
Days post-infection
R
RΔ4690c
RΔ4690c::4690c

Fig. S7. Bacterial burden of Δ*MAB_4690c* in BALB/c mice. **(a)** MALDI-MS spectra in positive mode of polar lipids from R (blue) show two signals at m/z 1392 (GL8Pa) and 1750 (GL8Pb) that were absent in R△*4690c* (red) and R△*MAB_4691c* (green). **(b-d) BALB/c** mice (n=5 to 11) were intravenously infected with 10^6 bacteria of *M. abscessus* R, R△*4690c*, or R△*4690c::4690c* and sacrificed at days 1, 15, and 30 post-infection. Lungs (**b**), spleen (**c**), and liver (**d**) were collected, homogenized, diluted, and plated on VCA3 agar plates. CFU counts were performed after 5 days of incubation at 37 °C. At day 30, p=0.0045 (RΔ4690c vs. RΔ4690c::4690c) in lungs; p=0.0018 (R vs. RΔ4690c) and p< 0.0001 (RΔ4690c vs. RΔ4690c::4690c) in spleen; p=0.029 (R vs. RΔ4690c) and p=0.0076 (RΔ4690c vs. RΔ4690c::4690c) in liver. Each dot in the scatter plots represents an individual mice represented with median with interquartile range. Results are expressed as mean ± SD. A significant diminution of the R△*4690c* bacterial loads was observed at 30 dpi, respectively to the R and R△*4690c::4690c* strains. ∗p<0.05; ∗∗p< 0.01; ∗∗∗∗ p< 0.0001. Source Data are provided as a Source Data file.

Table S1: Strains used in this study. A detailed compendium of *Mycobacterium abscessus* strains employed in this study, including the wild-type reference strain, gene-disrupted mutants, genetically complemented derivatives, and variants expressing fluorescent markers. Each strain is precisely characterized with a comprehensive description and its respective source or reference.

	**Description**	**Source**
M. abscessus CIP104536^T Rough strain (R)	Identifier: ATCC19977^T	Laboratoire de Référence des Mycobactéries (IP, France)
CIP R Δ*MAB_4690c*	CIP R in which the native *MAB_4690* is deleted.	This work
CIP R Δ*MAB_4690c*::HSP60, *MAB_4690c*	Kan^R derivative of CIP R Δ*MAB_4690* containing a copy of *MAB_4690* integrated into the attB site.	This work
CIP R Δ*MAB_4691c*	CIP R in which the native *MAB_4691* is deleted.	This work
CIP R::pMSP12, tdTomato	Hyg^R derivative of CIP R, expresses tdTomato	This work
CIP R Δ*MAB_4690c::* pMSP12, tdTomato, HSP60, *MAB_4690c*	Kan^R and Hyg^R derivative of CIP R Δ*MAB_4690* containing a copy of *MAB_4690* integrated into the attB site. Expresses tdTomato.	This work

Table S2. Summary of ^{1}H and ^{13}C chemical shifts and coupling constants from all amino-acids. Underlined values indicate confirmed correlations in **Fig. S4G-H** for *para-O*-methyl tyrosine and **Fig. S5A** for C-term carboxymethyl threonine (**upper panel**). Summary of ^{1}H and ^{13}C chemical shifts and coupling constants from α rhamnopyranosyl residues I and II (Rha-I and Rha-II). Spin systems are deduced from $^{1}H/^{1}H$ COSY spectra (**Fig. S3B**). Underlined values indicate acylated and methoxylated positions for Rha-I and Rha-II, respectively, which were confirmed by $^{1}H/^{13}C$ HMBC and/or $^{1}H/^{1}H$ NOESY correlations (**lower panel**).

Amino acids	δ (ppm)	NH	CO	$CH_α$	$CH_β$	$CH_{γ1}$	$CH_{γ2}$	$CH_{δ1}$	$CH_{δ2}$	$CH_ε$	$CH_ζ$	CH_3-O
Thr-1	^{1}H	7,64	-	4,150 *(d;6)*	3,985 *(m)*	1,218 *(d;6,2)*	-	-	-	-	-	-
	^{13}C	-	171,95	59,82	67,99	19,85	-	-	-	-	-	-
Ala-2	^{1}H	8,19	-	4,376	1,238 *(d;7)*	-	-	-	-	-	-	-
	^{13}C	-	173,99	49,29	16,85	-	-	-	-	-	-	-
Phe-3	^{1}H	8,06	-	4,387	3,119/2,944	-	-	7,15	-	7,23 *(Dd;7,6)*	7,17	-
	^{13}C	-	173,61	56,16	37,14	136,9	-	129,54	-	128,89	127,31	-
Ile-4	^{1}H	7,46	-	3,870 *(d;6,2)*	1,63	0,870/0,814	0,52 *(d;6,9)*	0,64 *(t;7,4)*	-	-	-	-
	^{13}C	-	173,15	59,5	35,86	24,26	15,5	11,2	-	-	-	-
Tyr-5	^{1}H	7,85	-	4,373	3,093/2,924	-	-	7,14	-	6,77 *(d;8,7)*	-	3,728
	^{13}C	-	172,69	56,38	35,96	n.d.	-	130,48	-	114,19	158,89	55,31
Leu-6	^{1}H	7,83	-	4,27 *(Dd;4,5/10,7)*	1,58/1,54	1,153	-	0,77 *(d;6,5)*	0,825 *(d;6,7)*	-	-	-
	^{13}C	-	174,17	52,62	40,48	24,51	-	21,5	23,18	-	-	-
Thr-7	^{1}H	7,73	-	4,430 *(d;3,6)*	4,2 *(m)*	1,154 *(d;6,3)*	-	-	-	-	-	-
	^{13}C	-	171,3	58,74	67,77	19,53	-	-	-	-	-	-
Thr-8	^{1}H	8,03	-	4,600 *(d;5,8)*	4,1 *(m)*	1,185 *(d;6,1)*	-	-	-	-	-	3,717
	^{13}C	-	171,11	57,35	72,17	15,55	-	-	-	-	-	52,718

Monosaccharides	δ (ppm)	Positions							
		1	2	CH_2-CO	CH_3-O	3	4	5	6
Rha-I	^{1}H *(m; $^{3}J_{Hn/Hn+1}$)*	4,77 *(d;1,5)*	4,98 *(Dd;3,3)*	2,35/2,31		3,81 *(Dd;9,7)*	3,42 *(T;9,7)*	3,612	1,255 *(d;6,1)*
	^{13}C *($^{1}J_{C/H}$)*	94,73 *(170)*	72,6	34,5		76,1	72,82	69,7	17,7
Rha-II	^{1}H *(m; $^{3}J_{Hn/Hn+1}$)*	5,1 *(d;1,2)*	3,502 *(Dd;3,5)*		3,42	3,563 *(Dd;9,7)*	3,277 *(T; 9,6)*	3,529	1,206 *(d;6,1)*
	^{13}C *($^{1}J_{C/H}$)*	99,35 *(169)*	80,77		58,96	71,21	73,25	69,38	17,6
