# Supplementary material for: A glycosylated lipooctapeptide promotes uptake and growth of Mycobacterium abscessus in the host
Source: Nat Commun. 2025 Apr 8;16:3326. doi: 10.1038/s41467-025-58455-5 (PMC11978893; doi:10.1038/s41467-025-58455-5)
Supplement: Supplementary file 2 — Description of Additional Supplementary Files [file 41467_2025_58455_MOESM2_ESM.pdf]

Description of Additional Supplementary Files

Data S1 a: Genes involved in GPL production in *M. smegmatis* and their orthologs at two loci in *M. abscessus*

Data S1 b: Genomic GPL loci in Msmeg

Data S1 c: Mabs ATCC 1st GPL locus

Data S1 d: Mabs ATCC 2nd GPL locus

Data S1 e: GenBank annotation detail of genes present at the GPL biosynthetic gene cluster in *Mycobacterium smegmatis*. The genes of the NRPS enzymes responsible for the biosynthesis of non-ribosomal peptides are highlighted orange.

Data S1 f: GenBank annotation detail of genes present at locus 1 of GPL biosynthetic gene cluster in *Mycobacterium abscessus* ATCC 19977. The genes of the NRPS enzymes responsible for the biosynthesis of non-ribosomal peptides are highlighted orange.

Data S1 g: GenBank annotation detail of genes present at locus 2 of GPL biosynthetic gene cluster in Mycobacterium abscessus ATCC 19977. The genes of the NRPS enzymes responsible for the biosynthesis of non-ribosomal peptides are highlighted orange.

Data S1 h: Abbreviations

Data S1 i: i*n silico* prediction of ubiquitous and species specific GPL loci on available mycobacterial complete genomes
