# Supplementary material for: A glycosylated lipooctapeptide promotes uptake and growth of Mycobacterium abscessus in the host
Source: Nat Commun. 2025 Apr 8;16:3326. doi: 10.1038/s41467-025-58455-5 (PMC11978893; doi:10.1038/s41467-025-58455-5)
Supplement: Supplementary file 4 — Reporting Summary [file 41467_2025_58455_MOESM4_ESM.pdf]

nature portfolio

Corresponding author(s): *Double-anonymous peer review submissions: write DAPR and your manuscript number here instead of author names.*

Last updated by author(s): *YYYY-MM-DD*

Reporting Summary

Nature Portfolio wishes to improve the reproducibility of the work that we publish. This form provides structure for consistency and transparency in reporting. For further information on Nature Portfolio policies, see our Editorial Policies and the Editorial Policy Checklist.

Statistics

For all statistical analyses, confirm that the following items are present in the figure legend, table legend, main text, or Methods section.

n/a	Confirmed	
☐	☒	The exact sample size (*n*) for each experimental group/condition, given as a discrete number and unit of measurement
☐	☒	A statement on whether measurements were taken from distinct samples or whether the same sample was measured repeatedly
☐	☒	The statistical test(s) used AND whether they are one- or two-sided<br>*Only common tests should be described solely by name; describe more complex techniques in the Methods section.*
☐	☒	A description of all covariates tested
☐	☒	A description of any assumptions or corrections, such as tests of normality and adjustment for multiple comparisons
☐	☒	A full description of the statistical parameters including central tendency (e.g. means) or other basic estimates (e.g. regression coefficient) AND variation (e.g. standard deviation) or associated estimates of uncertainty (e.g. confidence intervals)
☒	☐	For null hypothesis testing, the test statistic (e.g. *F*, *t*, *r*) with confidence intervals, effect sizes, degrees of freedom and *P* value noted<br>*Give P values as exact values whenever suitable.*
☒	☐	For Bayesian analysis, information on the choice of priors and Markov chain Monte Carlo settings
☒	☐	For hierarchical and complex designs, identification of the appropriate level for tests and full reporting of outcomes
☒	☐	Estimates of effect sizes (e.g. Cohen's *d*, Pearson's *r*), indicating how they were calculated

Our web collection on statistics for biologists contains articles on many of the points above.

Software and code

Policy information about availability of computer code

Data collection	none
Data analysis	Prism 10.2.2 software (Graphpad, USA)

For manuscripts utilizing custom algorithms or software that are central to the research but not yet described in published literature, software must be made available to editors and reviewers. We strongly encourage code deposition in a community repository (e.g. GitHub). See the Nature Portfolio guidelines for submitting code & software for further information.

Data

Policy information about availability of data

All manuscripts must include a data availability statement. This statement should provide the following information, where applicable:

- Accession codes, unique identifiers, or web links for publicly available datasets
- A description of any restrictions on data availability
- For clinical datasets or third party data, please ensure that the statement adheres to our policy

All data are available.

Research involving human participants, their data, or biological material

Policy information about studies with human participants or human data. See also policy information about sex, gender (identity/presentation), and sexual orientation and race, ethnicity and racism.

Reporting on sex and gender	N/A
Reporting on race, ethnicity, or other socially relevant groupings	N/A
Population characteristics	See reference 38 in the manuscript
Recruitment	See reference 38 in the manuscript
Ethics oversight	See reference 38 in the manuscript

Note that full information on the approval of the study protocol must also be provided in the manuscript.

Field-specific reporting

Please select the one below that is the best fit for your research. If you are not sure, read the appropriate sections before making your selection.

☒ Life sciences ☐ Behavioural & social sciences ☐ Ecological, evolutionary & environmental sciences

For a reference copy of the document with all sections, see nature.com/documents/nr-reporting-summary-flat.pdf

Life sciences study design

All studies must disclose on these points even when the disclosure is negative.

Sample size	Considering our study, our main goal was just to reveal the in vivo presence of the analysed compound GL8P during disease in CF patients, by using a true positive collection of sera (based on positive culture for M. abscessus, in sputum of CF patients) and compared it to a true negative collection of sera (culture negative sputum from CF patient, or culture positive sputum for other mycobacteria) (Microbiol Spectr.2022 Jun 29;10(3):e0019222. doi: 10.1128/spectrum.00192-22; J Cyst Fibros. 2022 Mar;21(2):353-360. doi: 10.1016/j.jcf.2021.08.019.)
Data exclusions	N/A
Replication	See published manuscripts regarding the serum collection (Microbiol Spectr.2022 Jun 29;10(3):e0019222. doi: 10.1128/spectrum.00192-22; J Cyst Fibros. 2022 Mar;21(2):353-360. doi: 10.1016/j.jcf.2021.08.019.)
Randomization	N/A
Blinding	N/A

Reporting for specific materials, systems and methods

We require information from authors about some types of materials, experimental systems and methods used in many studies. Here, indicate whether each material, system or method listed is relevant to your study. If you are not sure if a list item applies to your research, read the appropriate section before selecting a response.

Materials & experimental systems

n/a	Involved in the study	
☐	☒	Antibodies
☐	☒	Eukaryotic cell lines
☒	☐	Palaeontology and archaeology
☐	☒	Animals and other organisms
☒	☐	Clinical data
☒	☐	Dual use research of concern
☒	☐	Plants

Methods

n/a	Involved in the study	
☒	☐	ChIP-seq
☒	☐	Flow cytometry
☒	☐	MRI-based neuroimaging

Antibodies

Antibodies used	anti-CD43 antibody (Becton Dickinson; dilution 1:1000); Alexa Fluor 488-conjugated anti-mouse secondary antibody (Molecular Probes, Invitrogen)
Validation	Antibodies commonly used in our lab for immunofluorescence experiments

Eukaryotic cell lines

Policy information about cell lines and Sex and Gender in Research

Cell line source(s)	THP-1 immortalized monocytic cells
Authentication	Open Biol. 2016 Nov;6(11):160185. doi: 10.1098/rsob.160185; https://www.atcc.org/products/
Mycoplasma contamination	None
Commonly misidentified lines (See ICLAC register)	N/A

Animals and other research organisms

Policy information about studies involving animals; ARRIVE guidelines recommended for reporting animal research, and Sex and Gender in Research

Laboratory animals	C3HeB/FeJ and BALB/c mice
Wild animals	N/A
Reporting on sex	Female
Field-collected samples	N/A
Ethics oversight	Agreement n°783223; approved by MESRI with APAFIS#11465-2016111417574906v4

Note that full information on the approval of the study protocol must also be provided in the manuscript.

Plants

Seed stocks	N/A
Novel plant genotypes	N/A
Authentication	N/A
